# Prevalence of sero-markers and non-invasive assessment of liver cirrhosis in patients with Hepatitis B virus infection in Freetown, Sierra Leone: a cross-sectional study

**DOI:** 10.1186/s12876-021-01892-5

**Published:** 2021-08-09

**Authors:** Sulaiman Lakoh, Emmanuel Firima, Darlinda F. Jiba, Matilda N. Kamara, Wadzani Gashau, Gibrilla F. Deen, Olukemi Adekanmbi, George A. Yendewa

**Affiliations:** 1 Division of Infectious Diseases, Department of Internal Medicine, College of Medicine and Allied Health Sciences, University of Sierra Leone, Freetown, Switzerland; 2 Department of Medicine, University of Sierra Leone Teaching Hospitals Complex, Freetown, Switzerland; 3 Clinical Research Unit, Department of Medicine, Swiss Tropical and Public Health Institute, Basel, Switzerland; 4 University of Basel, Basel, Switzerland; 5 Department of Medicine, University of Maiduguri Teaching Hospital, Maiduguri, Nigeria; 6 Department of Medicine, University of Ibadan, Ibadan, Nigeria; 7 Department of Medicine, University College Hospital, Ibadan, Nigeria; 8 Department of Medicine, Case Western Reserve University School of Medicine, Cleveland, OH USA; 9 Division of Infectious Diseases and HIV Medicine, University Hospitals Cleveland Medical Center, Cleveland, MD USA; 10 Johns Hopkins Bloomberg School of Public Health, Baltimore, MD USA

**Keywords:** HBV, Liver fibrosis, Cirrhosis, Resource-limited settings, Sierra Leone

## Abstract

**Background:**

Hepatitis B virus (HBV) is a major global health problem. Although sub-Saharan Africa has a high proportion of the global burden of HBV, the epidemiology and clinical features of HBV in this region are poorly characterized, and access to diagnostic and treatment services remain limited.

**Methods:**

We conducted a retrospective study of HBV-infected children and adults of all age groups who were evaluated at public and private health facilities in Freetown, Sierra Leone between January 2017 and January 2019. We assessed their clinical presentation, HBV sero-markers, stages of liver disease, prevalence of cirrhosis by non-invasive tools, and the proportion of treatment eligible patients using the criteria recommended by the World Health Organization’s 2015 treatment guidelines for HBV. Logistic regression was used to identify predictors of liver cirrhosis.

**Results:**

163 HBV patients included in the study, with mean age 32.6 years and 65.0% (106) being males. Most (84.0%) were asymptomatic at presentation. The majority (69.9%) were classified as having HBeAg-negative chronic infection (or inactive HBsAg carrier phase), 24.5% were in the HBeAg-negative immune active phase, 3.1% had HBeAg positive hepatitis, and 2.5% were HBsAg negative. The median Aspartate aminotransferase to Platelet Ratio (APRI) and Fibrosis-4 (FIB-4) scores were 0.37 and 0.80, respectively. The prevalence of cirrhosis was 7.6% and 6.2%, estimated by the APRI and FIB-4 scores, respectively. About 20.0% of patients were eligible for treatment with antiviral agents. Based on APRI scores, the presence of any symptom [adjusted odds ratio (aOR) 20.0, 95% confidence interval (CI) (4.1–85.9); *p* < 0.001], elevated direct bilirubin [aOR 12.1, 95% CI (1.9–63.0); *p* = 0.003], and elevated total bilirubin [aOR 16.1, 95% CI (3.2–80.8); *p* = 0.001] were independent predictors of cirrhosis.

**Conclusion:**

Although most patients with HBV infection were asymptomatic, the prevalence of liver cirrhosis and proportion of patients requiring antiviral treatment were substantial. This small study from a hyperendemic setting in Sierra Leone suggests that routine population-based screening may increase early detection and linkage of HBV patients to care before development of complications. Larger studies are needed to confirm our findings.

## Introduction

Hepatitis B virus (HBV) infection is a major public health problem worldwide. The latest estimate in 2015 reported that there were 257 million people chronically infected with HBV globally [1]. Sub-Saharan Africa is one of the regions with the highest burden of HBV and together with the Western Pacific region account for two-thirds (68%) of all chronic HBV cases [1, 2]. In 2015, the World Health Organization (WHO) has set a priority agenda for the global elimination of viral hepatitis as public health threat by the year 2030 [3].

According to the WHO’s global estimates, less than 10% of HBV patients are diagnosed and therefore continue to transmit the virus to their close contacts [2]. Compared with high-income settings, the proportion of HBV diagnosed in low- and middle-income countries is relatively low (0.8% versus 18%, respectively) [1, 2]. Undiagnosed HBV may progress to liver cirrhosis and hepatocellular carcinoma, both of which caused over 1.4 million deaths globally in 2015 [1]. This is a major challenge in sub-Saharan Africa where the majority of patients with chronic HBV have no defined symptoms and mostly present with the complications of chronic HBV infection. Patients with HBV in sub-Saharan Africa are likely to die of hepatocellular carcinoma at a younger age (median 38.9 years), leading to significant economic losses in the region [2].

Although there are currently no nationally representative data on the prevalence and knowledge of HBV in Sierra Leone [4], several small studies from various demographic groups in the country have reported the prevalence of HBV as high as 8–22%, consistent with being a hyperendemic setting [4–11]. Despite the high HBV burden, however, the country has limited laboratory capacity to diagnose, treat and monitor people infected with HBV [12]. Currently, Sierra Leone does not have a comprehensive national policy plan to address the viral hepatitis epidemic. Moreover, the lack of adequate diagnostic resources to collect population-level data on the demographic and clinical characteristics of people infected with HBV is hindering prevention and control efforts in the country. Thus, it is essential to strengthen diagnostic services along the continuum of HBV care in order to meet the WHO’s 2030 viral hepatitis elimination goals [1–3].

The goal of HBV treatment is to inhibit viral replication and prevent the long-term complications of HBV infection. Current treatment guidelines recommend using a combination of non-invasive and invasive approaches in the diagnosis, clinical staging, management and prognostication of patients with HBV infection [13–17]. Liver biopsy is the gold standard for staging liver disease; however, the unavailability or prohibitive cost of liver biopsy and other advanced diagnostic tools such transient elastography (FibroScan) has warranted the use of alternative methods for staging HBV disease and monitoring treatment for HBV infected in Sierra Leone and most other resource-limited settings [13, 14]. The aspartate aminotransferase (AST) to platelet (PLT) ratio index (APRI) and the fibrosis-4 (FIB-4) scores are two of the most widely used tools in this regard. The APRI and FIB-4 scores have the advantages of being non-invasive, relatively inexpensive, easily computed from routine laboratory findings and have been validated in resource-limited settings [14]. Furthermore, both the APRI and FIB-4 scores provide intermediate to high sensitivity and specificity in predicting liver fibrosis [14–19], and are the tools recommended by the WHO for assessing and staging viral hepatitis infection in resource-limited settings lacking advanced diagnostic facilities [14].

In this study, we characterised the clinical presentation and patterns of HBV serum markers, estimated the burden of liver fibrosis and assessed eligibility for HBV treatment using non-invasive tools in a cohort of HBV infected patients in a hyperendemic setting in Sierra Leone for the first time.

## Methods

### Study design, population and setting

We conducted a retrospective study of newly diagnosed patients with HBV at various healthcare facilities in Sierra Leone who were referred for further management to both Connaught Hospital and Premium Medical Services (SL) Limited in Freetown, Sierra Leone between January 2017 and January 2019. Connaught Hospital is the national referral healthcare center. It is a 300-bed academic facility affiliated with the College of Medicine and Allied Health Sciences of the University of Sierra Leone and provides outpatients and in-patients services. Premium Medical Services is a private business entity in Freetown, Sierra Leone. The facility only provides outpatients medical services.

Despite the high burden of HBV in Sierra Leone, there are currently few viral hepatitis specialty clinics for the management of patients, and referrals specifically for HBV treatment are uncommon in this setting. Our study therefore included patients of all age groups who sought care for HBV at our facilities during the study period.

### Clinic and laboratory procedures

Opon referral, patients were assessed in accordance with the recommendations of the Sierra Leone National Treatment Guidelines for Viral Hepatitis B and C [13] and the WHO Guidelines for the Prevention, Care and Treatment of persons with Chronic Hepatitis B infection (2015), respectively [14].

All patients were evaluated for signs and symptoms of viral hepatitis. Complete blood count (white cell, hemoglobin and platelet count), basic metabolic panel (electrolytes, glucose, urea and creatinine), and liver function tests were determined using automated haematology and biochemistry analysers by Cypress Diagnostics, respectively. Human immunodeficiency virus (HIV) status was determined using the rapid test by SD Bioline HIV-1/2 3.0 (Standard Diagnostics, Inc) and while HBV and hepatitis C virus (HCV) status were determined using the Commercial testing kit Citest™ Diagnostics Inc (Canada), according to the manufacturer's instructions.

A complete HBV serological profile was further determined for all patients as follows: hepatitis B surface antigen (HBsAg), antibodies to HBsAg (HBsAb), HBV pre-core antigen (HBeAg), antibodies to HBeAg (HBeAb), and the total and IgM-specific antibodies (HBcAb and HBcAb IgM, respectively).

Chronic HBV status was classified according to the five phases of the natural history of chronic HBV infection, defined by the European Association for the Study of the Liver, as follows: (1) HBeAg-positive chronic infection, (2) HBeAg-positive chronic hepatitis, (3) HBeAg-negative chronic infection, (4) HBeAg-negative chronic hepatitis, and (5) HBsAg-negative phase [16].

The APRI and FIB-4 scores were used to estimate the prevalence of liver cirrhosis. The natural history of HBV co-infection with HIV, HCV or hepatitis D virus (HDV) differs significantly from that of HBV mono-infection, with accelerated progression to cirrhosis, end-stage liver disease and hepatocellular carcinoma [20–23]. To maintain homogeneity of the study population, we therefore removed co-infected patients from the analysis for the assessment of cirrhosis.

The APRI was calculated using the formula {AST (IU/L)/AST (Upper Limit of Normal) (IU/L)}/{Platelet Count (10^9^/L)} × 100. The following APRI thresholds were used to determine the stage of liver disease: APRI < 0.5, normal liver architecture; APRI 0.5–2.0, moderate to significant fibrosis; and APRI > 2.0, cirrhosis [14–19]. In accordance with the WHO 2015 hepatitis treatment guidelines, we used APRI > 2.0 as the threshold to determine treatment eligibility for HBV infection in settings with limited diagnostic facilities [14].

The FIB-4 score was calculated using the formula {Age (years) × AST (IU/L)}/{Platelet Count (10^9^/L) × [ALT (IU/L)]^1/2^}. A FIB-4 score < 1.45 was interpreted as normal liver architecture, FIB-4 score of 1.45–3.25 indicated moderate to significant fibrosis, while FIB-4 > 3.25 indicated cirrhosis [14–19].

### Data collection and definitions

Demographic, clinical and laboratory data are collected from the medical records of registered patients of all ages who have been diagnosed with HBV infection. Hepatitis B virus infection was defined as having a positive HBsAg result within 90 days of referral and HBV treatment-naïve. Alcohol use was defined as the consumption of > 20 g or 2 glasses of alcoholic beverages daily, while illicit drug use is defined as the use of any amount of marijuana, cocaine or injected heroin in the past 30 days.

### Statistical analysis

All data were computed in an excel spreadsheet and transferred into STATA version 16 (StataCorp LLC) and SPSS Version 25.0 (Armonk, NY; IBM Corp) for analysis. Normally distributed continuous variables were reported with means and standard deviation, while non-normally distributed continuous variables were reported with medians and interquartile range. Continuous laboratory values were appropriately reported using means or medians, and categorised based on standardized laboratory-specific reference values. Logistic regression models were used to assess predictors of cirrhosis based on the APRI and FIB-4 scores, adjusting for age, sex, laboratory parameters (direct and indirect bilirubin levels) and the presence of any clinical features or symptoms typically associated with cirrhosis (i.e., jaundice, abdominal swelling, fatigue or passage of dark urine). Variables which attained a *p* value of < 0.2 in the univariate analysis were included in the multivariate regression model. Associations were reported as crude (OR) and adjusted odds ratios (aOR) with 95% confidence intervals (CI). Differences were considered statistically significant when *p* was < 0.05.

### Ethical considerations

Ethics approval was obtained from the Sierra Leone Ethics and Scientific Review Committee of the Ministry of Health and Sanitation. Written informed consent was not required for this retrospective study as it has been waived by the Sierra Leone Ethics and Scientific Review Committee of the Ministry of Health and Sanitation. Data were anonymized and stored securely in a password protected device to ensure patient confidentiality.

## Results

### Demographic and serological characteristics

A total of 163 patients with HBV infection were included in the study. The majority (106/163, 65.0%) were males. The mean age of the patients was 32.6 years (standard deviation 11.1), and most (131/163, 80.4%) were aged 20–45 years and single (82/163, 50.3%). Most patients (114/163, 69.9%) were classified as having HBeAg-negative chronic infection (or inactive HBsAg carrier phase), 24.5% (40/163) were in the HBeAg-negative (or immune active) phase, 3.1% (5/163) had HBeAg positive hepatitis, and 2.5% (4/163) were HBsAg negative (spontaneous seroclearance). The HIV/HBV co-infection rate was 3.1% (5/163), whereas the HBV/HCV co-infection rate was 0.6% (1/163). Details of the demographic and serological characteristics are presented in Table 1**.**

**Table 1:** Demographic clinical and serological characteristics of patients with HBV (N = 163)

Patient characteristics	Frequency N (%)	HBV serology	Frequency N (%)
**Sex**		**Anti-HBs**	
Male	106 (65.0)	Yes	1 (0.6)
Female	57 (35.0)	No	162 (99.4)
**Age**		**HBeAg**	
< 10	1 (0.6)	Yes	5 (3.1)
10—19	12 (7.4)	No	158 (96.9)
20—30	66 (40.5)	**Anti-HBe**	
31—45	65 (39.9)	Yes	154 (94.5)
> 45	19 (11.7)	No	9 (5.5)
**Marital status**		**Anti-HBc IgM**	
Single	82 (50.3)	Yes	7 (4.3)
Married	74 (45.4)	No	156 (95.7)
Divorced	2 (1.2)	**Anti-HBc IgG**	
Widowed	4 (2.5)	Yes	153 (93.9)
Separate	1 (0.6)	No	10 (6.1)
**Occupation**			
Unemployed	15 (9.2)	**HBV/HCV**	1 (0.6)
Student	45 (27.6)		5 (3.0)
Informal sector	43 (26.4)	**HBV/HIV**	
Formal sector	57 (35.0)		
Retired	3 (1.8)	**Classification of chronic HBV**	
**Clinical features**		HBeAg-positive chronic infection	4 (2.5)
Asymptomatic	137(84.0)	HBeAg-positive chronic hepatitis	1 (0.6)
Symptomatic	26(16.0)	HBeAg-negative chronic infection	114 (69.9)
Jaundice	14(8.6)	HBeAg-negative chronic hepatitis	40 (24.5)
Passage of dark urine	12(7.4)	HBsAg-negative chronic hepatitis	4 (2.5)
Abdominal swelling	8(4.9)		
Fatigue	7(7.3)		

### Clinical presentation and baseline laboratory characteristics

Most of the patients were asymptomatic at the time of initial evaluation (137, 84.0%), with 26 (16.0%) showing clinical features (Table 1). Among the 26 patients with clinical features, jaundice was the most common complaint (14/26, 54.0%), followed by passage of dark urine (12/26, 46.0%), abdominal swelling (7/26, 27.0%), and fatigue (8/26, 3%).

Table 2 displays the laboratory parameters of HBV patients. Serum protein was elevated in 55.2% (90/163) of the patients. Among the liver enzymes tested, 32.5% (55/163), 31.3% (51/163), 31.3% (51/163), and 25.2% (41/163) patients had elevated alkaline phosphatase (ALP), AST, gamma-glutamyl transferase (GGT), and ALT, respectively. Eighteen (11.0%) of patients had ALT values at least twice the upper limit of normal (≥ 2 × ULN).

**Table 2:** Laboratory parameters of patients with HBV

Laboratory parameter	Median (IQR) or Mean (SD) value of assay result	Patients (%) with normal or deranged assay result	Reference values^#^
Alanine aminotransferase (IU/L)	28 (21–41)	↑ 41 (25.2)	< 40 IU/L
		↔ 122 (74.8)	
		***18 (11.0)***^***§***^	
		***145 (89.0)***^***¶***^	
Aspartate aminotransferase (IU/L)	27 (19–45)	↑ 51 (31.3)	< 38 IU/L
		↔ 112(68.7)	
Alkaline phosphatase (IU/L)	84 (52–142)	↑ 53 (32.5)	26–117 IU/L
		↔ 100 (61.3)	
		↓ 10 (6.1)	
Total bilirubin (mg/dL)	0.7 (0.5–1.1)	↑ 43 (26.4)	Up to 1.0 mg/dL
		↔ 120 (73.6)	
Direct bilirubin (mg/dL)	0.20 (0.10–0.30)	↑ 53 (32.5)	Up to 0.25 mg/dL
		↔ 110 (67.5)	
Gamma glutamyl transferase (IU/L)	30 (22–64)	↑ 51 (31.3)	11–50 IU/L
		↔ 109 (66.9)	
		↓ 3 (1.8)	
Total protein (mg/dL)	59.0 (7.0–69.0)	↑ 90 (55.2)	6.5–8.7 mg/dL
		↔ 59 (36.2)	
		↓ 14 (8.6)	
Albumin (mg/dL)	22.7 ± 18.6*	↑ 86 (52.8)	3.5–5.3 mg/dL
		↔ 55 (33.7)	
		↓ 22 (13.5)	
Platelets (× 10^9^/L)	213 ± 78*	↑ 1 (0.6)	150–450 (× 10^9^/L)
		↔ 132 (81.0)	
		↓ 30 (18.4)	

IQR, interquartile range. SD, standard deviation. *Mean and standard deviation reported. ↑ = Elevated. ↔  = Normal. ↓ = Reduced. § = more than 2 times-, ¶ = less than 2 times upper limit of normal. # = Reference values

### Staging liver disease by liver ultrasound imaging, APRI and FIB-4 scores

Of the 163 patients evaluated for HBV, only 43.6% (71/163) had abdominal ultrasound scan performed (Table 3). Of these, the majority (69.0%, 49/71) had a normal appearing liver, 22.6% (16/71) had features of cirrhosis, 5.6% (4/71) had features suggestive of a hepatoma (confirmed by abdominal CT scan), and 2.8% (2/71) had fatty liver.

**Table 3:** Non-invasive staging of liver fibrosis by APRI and FIB-4 scores and ultrasound imaging

Diagnostic Tool	Frequency (%) or Median (IQR)	Clinical staging
**AST to Platelet Ratio Index** *(N* = *129)*		
Median (IQR)	0.36 (0.24–0.60)	
< 0.5	87 (67.4)	Normal liver architecture
0.5–2.0	30 (23.3)	Moderate to significant fibrosis
> 2.0	12 (7.6)	Cirrhosis
**Fibrosis-4 Score** *(N* = *134)*		
Median (IQR)	0.80 (0.48–1.27)	
< 1.45	106 (79.1)	Normal liver architecture
1.45–3.25	19 (14.2)	Moderate to significant fibrosis
> 3.25	9 (6.2)	Cirrhosis
**Liver ultrasound imaging** *(N* = *71)*		
	49 (69.0)	Normal liver architecture
	16 (22.6)	Cirrhosis
	4 (5.6)	Hepatoma
	2 (2.8)	Fatty liver

IQR, interquartile range

We calculated the APRI and FIB-4 scores after removing the 5 patients with HBV/HIV co-infection and the one patient with HBV/HCV co-infection from the analysis (Table 1). One hundred and twenty-nine HBV patients had laboratory results to compute the APRI score (Table 3). The median APRI score was 0.37 (IQR 0.25–0.57). Based on the APRI thresholds used for staging liver disease, 67.4% (87/129) were classified as having normal liver architecture (APRI < 0.5), 23.3% (30/129) had moderate to significant fibrosis (APRI 0.5–2.0), while 7.6% (12/129) were classified as having cirrhosis (APRI > 2.0).

One hundred and thirty-four patients had laboratory results to compute the FIB-4 score (Table 3). The median FIB-4 score was 0.8 (IQR 0.55–1.3). Using the FIB-4 thresholds for staging liver disease, 79.1% (106/134) were classified as having normal liver architecture (FIB-4 < 1.45), 14.2% (19/134) had moderate to significant fibrosis (FIB-4 1.45–3.25), and 6.2% (9/134) were classified has having cirrhosis.

### Predictors of liver cirrhosis

In the multivariate analysis of APRI scores, presence of one or more clinical feature or symptom typically associated with liver cirrhosis [aOR 20.0, 95% CI (4.1–85.9); *p* < 0.001], elevated total bilirubin [aOR 16.1, 95% CI (3.2–80.8); *p* = 0.001] and elevated direct bilirubin [aOR 12.1, 95% CI (1.9–63.0); *p* = 0.003] strongly predicted liver cirrhosis (Table 4). However, none of the tested variables reached statistical significance in predicting liver cirrhosis using FIB-4 scores.

**Table 4:** Multivariable evaluation of APRI sore (> 2.0) and FIB-4 score (> 3.25) to predict liver cirrhosis, adjusted for age and sex

Variables	APRI Score			FIB-4 Score		
	Crude OR 95% (CI)	Adjusted OR 95% (CI)	*p* Value	Crude OR 95% (CI)	Adjusted OR 95% (CI)	*p* Value
**Clinical features or symptoms of liver cirrhosis**						
None present	Ref		** < 0.001**	Ref		
One or more present	19.8 (4.8–81.7)	20.0 (4.1–85.9)		2.0 (0.5–8.5)	–	0.298
**Direct bilirubin**						
Not elevated	Ref		**0.003**	Ref		
Elevated	11.0 (2.0–53.0)	12.1 (1.9–63.0)		1.8 (0.5–6.2)	–	0.348
**Total bilirubin**						
Not elevated	Ref		**0.001**	Ref		
Elevated	15.6 (3.0–75.0)	16.1 (3.2–80.8)		3.7 (1.1–13.3)	3.3 (0.9–11.6)	0.065

### Proportion of eligible patients for treatment with antiviral therapy

Using the criteria described in the WHO 2015 treatment guidelines for HBV [14], 19.6% (32/163) of patients with HBV referred to our facilities were eligible for treatment initiation with antiviral therapy (Table 5). Several patients fulfilled one or more eligibility criteria for treatment with antiviral therapy. Treatment was indicated in 7 patients with either an elevated ALT that was 2 × ULN or sonographic evidence of cirrhosis. A positive HIV status in the absence of other indications was reported in 4 patients with chronic HBV (Table 5).

**Table 5:** Estimate of patients with HBV requiring antiviral therapy and their indications

Sonographic evidence of advanced fibrosis or cirrhosis	APRI > 2.0	FIB-4 > 3.25	HIV positive	ALT > 2 × ULN	Frequency
–	–	–	–	X	7
X	–	–	–	–	7
–	X	–	–	X	4
–	–		X	–	4
X	X		–	X	2
X	X	X		X	2
X	–	X	–	–	1
X	–	–	–	X	1
X		X		X	1
–	–	X	–	X	1
X	–	X	–	–	1
X	–	–	X	–	1
Total					32

ALT, Alanine aminotransferase; ULN, upper limit of normal; X = Treatment indicated; (–) = treatment not indicated

## Discussion

This is the first study from Sierra Leone to describe the clinical presentation and patterns of HBV sero-markers, estimate the prevalence of liver cirrhosis, and assess the eligibility for antiviral therapy of newly diagnosed HBV infected patients. The majority of patients (84.0%) referred to our facilities had no symptoms or signs suggestive HBV infection at the time of first evaluation. The absence of symptoms in chronically infected HBV patients may result in delayed access to treatment, care and support services. This may have several implications in the response against the HBV epidemic in the country. Firstly, asymptomatic patients with late presentation to care may continue to transmit the virus to their close contacts in the community. Secondly, patients with chronic HBV not receiving treatment have a heightened risk (15–40%) of progressing to liver cirrhosis, hepatocellular carcinoma or end stage liver disease, especially in the presence of other risk factors such as excess alcohol consumption or illicit drug intake [24, 25]. The delay in seeking diagnostic and treatment services for HBV infection is not unique to Sierra Leone and is reflective of current global trends, as an estimated 90% of people infected with HBV globally remain undiagnosed [2]. Thus, our study highlights missed opportunities that could benefit from public health strategies promoting early detection and linkage of HBV infected patients to treatment services in Sierra Leone.

At presentation, about 3.1% of patients were classified as having HBeAg-positive hepatitis, while 2.5% had already spontaneously achieved HBsAg seroclearance (i.e., HBsAg negative). In contrast, however, the majority of patients (nearly 70%) were classified as having HBeAg-negative chronic infection, similar to a report from Cameroon [26]. Previously known as the immune controlled or inactive HBsAg carrier stage, this phase may follow seroconversion from HBeAg to anti-HBe antibody, and is characterized by a robust host immune response, leading to low or undetectable viremia (i.e., HBV DNA < 2000 IU/mL) and minimal liver damage (persistently normal markers of necroinflammation). Treatment with antivral drugs is generally not warranted during this stage, and studies have shown that the majority of patients (70%-80%) tend to have a favorable prognosis, with low risk of progression to cirrhosis or hepatocellular carcinoma. However, it is estimated that patients in this phase have 4%-20% risk of reversion back to HBeAg positive hepatitis [27]. Regular clinical monitoring and surveillance are therefore recommended (typically every 6 months) to avoid the serious sequalae of chronic HBV infection [14–17].

Furthermore, about 25% of our patients were classified in the HBeAg-negative chronic hepatitis phase (also known as the immune-active phase), which is characterised by low level viremia due to ongoing relocation (i.e., HBV DNA > 2000 IU/mL) and mildly elevated markers of hepatic necroinflammation. This phase has been associated with a 10%-20% risk of HBV reactivation [28]. Unlike the HBeAg-negative chronic infection phase, current guidelines recommend antiviral therapy and more frequent clinical and laboratory monitoring (typically every 3–4 months), as the HBeAg-negative chronic hepatitis phase confers a substantially higher risk of progression to cirrhosis and hepatocellular carcinoma [14–17, 28].

Using the thresholds APRI > 2.0 and FIB-4 > 3.25, an estimated 7.6% and 6.2% of our patients, respectively, met the criteria for cirrhosis. In comparison, ultrasound imaging overestimated the prevalence of cirrhosis (22.6%) in the 71 patients that were able to afford the cost of the test. A systemic review and meta-analysis by Surial et al. [29] reported a pooled cirrhosis prevalence of 6.1% (n = 3204) in sub-Saharan Africa, using a combination of non-invasive assessments including transient elastography, APRI score and FibroTest. The prevalence of cirrhosis in our study using the APRI and FIB-4 scores are in agreement with Surial et al. [29] and with other reports from the West Africa region which have estimated the prevalence of cirrhosis among HBV mono-infected individuals as ranging from 0% (n = 26) in prisoners in the Ivory Coast [30] to 7.3% (n = 300) in blood donors in the Gambia [31].

The APRI score appeared to perform better than the FIB-4 score at predicting cirrhosis in our study. Using the APRI score, the predictors of cirrhosis were presence of any symptoms and elevations in either direct or total bilirubin, adjusted for age and sex. Direct bilirubin as an independent predictor of cirrhosis was a noteworthy finding. Various prognostic tools used to predict liver disease outcomes including the Child–Pugh score and Model for End-stage Liver Disease (MELD) score incorporate total bilirubin into their models [15, 16]. The pathophysiology of direct and indirect bilirubinemia differ, and because direct bilirubin levels are better reflective of hepatic synthetic activity, it has been postulated that incorporating direct bilirubin rather than total bilirubin would increase the prognostic performance of models predicting liver disease outcomes, especially in liver cirrhosis [32]. Using direct bilirubin, (n = 983), Lee et al. [33] recently demonstrated the superiority of a new prognostic model known as DiBIC (Direct Bilirubin, INR, and Creatinine) over the MELD score in predicting 6-month mortality in patients with liver cirrhosis.

Not all patients with HBV require treatment with antiviral drugs. Only about 20% of patients in our study were eligible for initiation of antiviral treatment based on the criteria set by the 2015 WHO treatment guidelines for HBV [14]. Important considerations influencing decision to treat an individual patient include age, phase of chronic HBV, co-infection with HIV or HDV, presence of complications such as cirrhosis, and special populations including pregnant women, transplant patients and other immunocompromised patients on immunosuppressive therapy who are at high risk of HBV reactivation [15, 16]. Estimates of proportion of HBV-infected patients who require treatment with antiviral therapy should be considered by public health experts and policy makers for program planning and budgeting, as Sierra Leone and other endemic countries work towards achieving the global viral hepatitis elimination goals by 2030.

### Limitations

Our study had limitations and strengths that are worth mentioning. A major limitation was the small sample size, which may make the generalizability of the study findings difficult. Secondly, due to limited diagnostic capacity and cost constraints, we were unable to offer all patients in this low income setting a complete initial assessment with all the baseline laboratory (especially quantification of HBV DNA by PCR) and imaging tests recommended by current treatment guidelines; thus, classification of phases of chronic HBV, staging of liver disease, risk stratification, and treatment eligibility estimates were approximations. However, whereas previous studies from Sierra Leone have focused on describing the prevalence and risk factors of HBV, a key strength of our study is that contributes new knowledge on the presentation, clinical features, predictors of adverse outcomes such as cirrhosis, and estimates of proportion of patients eligible for treatment with antiviral drugs—all of which are important considerations in crafting and implementing an effective control and prevention program for tackling HBV in this endemic setting.

## Conclusion

In summary, most patients with HBV infection (84%) were asymptomatic at presentation. The majority (70%) were in the HBeAg negative chronic infection (or inactive HBsAg carrier) phase of infection, while only 3.1% of patients had HBeAg positive hepatitis. A substantial proportion of patients (6.2–7.6%) had liver cirrhosis based on non-invasive tools (APRI and FIB-4), and about 20% of patients were eligible for treatment with antiviral agents. Presence of any symptom and elevated direct and total bilirubin levels independently predicted cirrhosis. Larger studies are needed to improve the generalizability of our findings. Despite the small sample size, however, our study may have implications for HBV prevention and control efforts in Sierra Leone.

## Abbreviations

ALP: Alkaline phosphatase
ALT: Alanine transaminase
APRI: AST to platelet ratio index
AST: Aspartate transaminase
DNA: Deoxyribonucleic acid
FIB-4: Fibrosis-4
GGT: Gamma-glutamyl transferase
HBeAg: Hepatitis B precore antigen
HBeAb: Anti-body to hepatitis B precore antigen
HBsAg: Hepatitis B surface antigen
HBsAb: Antibody to hepatitis B surface antigen
HBcAg: Hepatitis B core antigen
HBcAb: Antibody to hepatitis B core
HCV: Hepatitis C virus
PLHIV: People living with HIV
TB: Tuberculosis
WHO: World Health Organization
ULN: Upper limit of normal

## Declarations

### Availability of data

The data supporting this article is available in the repository of University of Sierra Leone and will be made easily available on request to the corresponding author when required.
